# Application of clinical blood metabogram for diagnosis of early-stage Parkinson’s disease: a pilot study

**DOI:** 10.3389/fmolb.2024.1407974

**Published:** 2024-08-14

**Authors:** Petr G. Lokhov, Oxana P. Trifonova, Elena E. Balashova, Dmitry L. Maslov, Michael V. Ugrumov, Alexander I. Archakov

**Affiliations:** ^1^ Laboratory of Mass Spectrometric Metabolomic Diagnostics, Institute of Biomedical Chemistry, Moscow, Russia; ^2^ Laboratory of Neural and Neuroendocrine Regulations, Koltzov Institute of Developmental Biology of the Russian Academy of Sciences, Moscow, Russia

**Keywords:** metabolomics, clinical blood metabogram, Parkinson’s disease, diagnostics, mass spectrometry, blood plasma, clinical metabolomics, personalized metabolomics

## Abstract

In terms of time, cost, and reproducibility of clinical laboratory tests, a mass spectrometric clinical blood metabogram (CBM) enables the investigation of the blood metabolome. Metabogram’s components provide clinically relevant information by describing related groups of blood metabolites connected to humoral regulation, the metabolism of lipids, carbohydrates and amines, lipid intake into the organism, and liver function. For further development of the CBM approach, the ability of CBM to detect metabolic changes in the blood in the early stages of Parkinson’s disease (PD) was studied in this work. In a case-control study (n = 56), CBM enabled the detection of the signature in blood metabolites related to 1–2.5 clinical stages of PD, according to the modified Hoehn and Yahr scale, which is formed by alterations in eicosanoids, phospholipids and, presumably, in the butadione metabolism. The CBM component-based diagnostic accuracy reached 77%, with a specificity of 71% and sensitivity of 82%. The research results extend the range of disorders for which CBM is applicable and offer new opportunities for revealing PD-specific metabolic alterations and diagnosing early-stage PD.

## 1 Introduction

Parkinson’s disease (PD), which usually affects elderly people, is the second most prevalent neurodegenerative condition of the central nervous system. Due to the aging population, the incidence of PD has substantially grown. Since the first description of PD by James Parkinson in 1817 (Parkinson, 2002), the exact mechanism causing this disease is still unknown. Hallmarked dopaminergic neurons that are destroyed in the substantia nigra and the formation of Lewy bodies that are largely made of fibrillar α-synuclein are pathological characteristics of PD (Tansey and Goldberg, 2010). Genetic studies of familial PD have identified mutations in individual genes in monogenic PD. In particular, mutations leading to the development of PD are localized in the genes encoding α-synuclein, dardarin, vacuolar protein sorting-associated protein 35, parkin ligase, DJ1 deglycase, and acid β-glucosidase (Ross, 2013; Deng et al., 2018).

There are evidences that oxidative damage and mitochondrial dysfunction lead to a cascade of events and ultimately contribute to the degeneration of dopaminergic neurons (Rani and Mondal, 2020). Other studies demonstrated that apoptosis plays a substantial role in neurological disorders (Tompkins et al., 1997). Recent studies have linked astrogliosis to the development of PD (Heo et al., 2020).

The neuroinflammatory theory appears to be the most plausible of the potential causes of PD (Tansey and Goldberg, 2010; Gelders et al., 2018). The inflammatory process, which is a protective mechanism against various types of damage, when prolonged, enhances the progression of neurodegeneration (Snyder et al., 2017). A role of neuroinflammation in the pathology of PD was demonstrated in a large number of studies, indicating that neuroinflammatory processes may play a causative role in the development of PD (Salama et al., 2020).

Several other factors causing PD, such as reduced Parkin activity, altered metabolism, aberrant epigenetics, exposure to toxins, telomere shortening, or protein misfolding, were reported in a number of studies (Le et al., 2013; Scheffold et al., 2016; Wen et al., 2016; Rokad et al., 2017; Meng et al., 2020). According to some studies, PD can be classified as a prion-like disease (Olanow and Brundin, 2013).

Due to the still unclear etiology and pathogenesis of PD, the identification of biomarkers for its diagnosis is challenging and has not been successful yet (Chen-Plotkin et al., 2018). In this situation, the omics technologies, which enable measuring the diversity of a biologic system’s molecules in a single-run analysis (e.g., DNA sequencing in genomics, protein identification technologies in proteomics, and profiling of low-molecular-weight substances in metabolomics), may be helpful (Omenn et al., 2012). Among the omics sciences, metabolomics is the most promising for supplying useful information for disease diagnostics because metabolites form molecular phenotypes directly reflecting physiological and pathological situations in organisms. Thus, in metabolomics studies of blood, the diagnostic accuracy of diseases often reaches 90%–95% (Trifonova et al., 2013). Such results stimulate the introduction of metabolomics technologies in medicine for the diagnosis of difficult-to-diagnose diseases, including PD, the etiology and pathogenesis of which is often associated with low-molecular substances.

**FIGURE 1:**
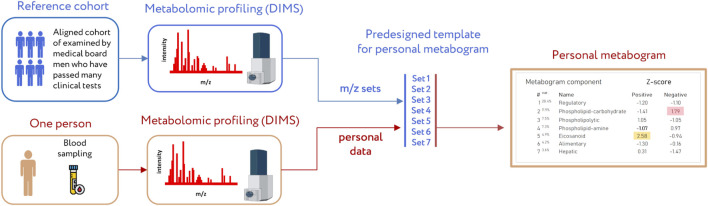
Workflow for producing a clinical blood metabogram. Sampled blood, after sample preparation in order to separate the metabolome fraction, is subjected to direct-infusion mass spectrometry (DIMS). The resulting mass peaks are aligned with the predefined sets of mass spectrometric peaks corresponding to the components of the metabogram (predesigned template of personal metabogram). Mass peak intensities are converted into Z-scores and averaged to obtain metabogram component values showing the state (normal, upregulated, or downregulated) of the blood metabolome (i.e., clinically relevant information). Adapted from (Lokhov et al., 2023b).

A clinical blood metabogram (CBM), a new personalized metabolomics approach that is a simplified single-subject (N-of-1) metabolomics analysis, was recently introduced (Lokhov et al., 2023b). Direct-infusion mass spectrometry (DIMS), principal component analysis (PCA), and metabolite set enrichment analysis (MSEA) were used to develop the CBM. The metabogram avoids the complexity of each N-of-1 metabolomics study and is characterized by rapid execution, simple data processing, high reproducibility, and uncomplicated result interpretation, which should make it easier to apply CBM in the clinic in the laboratory-developed test (LDT) format (Figure 1). An LDT is a specific kind of diagnostic test that is created, produced, and utilized in a single laboratory (Sharfstein, 2015; FDA., 2018; Genzen, 2019; Schreier et al., 2019) that is commonly used to implement omics tests.

The blood metabolome groups that deal with humoral control, lipid-carbohydrate and lipid-amine metabolism, eicosanoids, amino acids, lipid intake into the body, and liver function are presented in the CBM that makes it clinically valuable. The main objective of this study is to examine the metabogram’s clinical potential in relation to revealing metabolic features and diagnosing early PD. To do this, the blood metabolome of patients with PD 1–2.5 stages, as measured by the modified Hoehn and Yahr scale, was examined using the CBM.

## 2 Materials and methods

### 2.1 Blood samples

Samples of blood plasma used in this study were taken from a previously published study, where study participants (n = 56) were recruited at the Republican Clinical Diagnostic Centre of Extrapyramidal Pathology and Botulinum Therapy (Kazan, Russia) (Balashova et al., 2018). Briefly, study cohort included untreated PD patients at 1–2.5 stages according to modified Hoehn and Yahr scale (stage 1 – unilateral involvement only; stage 1.5 – unilateral and axial involvement; stage 2 – bilateral involvement without impairment of balance; stage 2.5 – mild bilateral disease with recovery on pull test) (Goetz et al., 2004) and controls without neurodegenerative diseases. The following exclusion criteria were used for PD patients and control subjects: severe systemic disease, stroke, brain surgery, Alzheimer’s disease or any other medical history central nervous system disease, chronic renal failure, systemic infections, malignancy, cardiac or hepatic dysfunction, and autoimmune disease. Informed consent was obtained from all subjects involved in the study. The study was conducted in accordance with the Declaration of Helsinki, and approved by the Institutional Ethics Committee of Koltzov Institute of Developmental Biology of Russian Academy of Sciences (protocol code 55 and date of approval 9 December 2021).

### 2.2 Mass spectrometry analysis of blood samples

The same equipment and materials were used as in the previously reported study (Balashova et al., 2018), including venous blood sampling, sample preparation, mass spectrometer analysis, mass spectra processing, and mass list processing (alignment, standardization, and normalizing).

Blood samples were taken from the vein before the morning meal. Samples (3 mL) were placed into glass tubes containing K_2_EDTA (BD Vacutainer; Becton, Dickinson and Company, Franklin Lakes, NJ, United States) and centrifuged within 15 min of blood collection at 1,600 × g and room temperature. The resultant blood plasma was subdivided into aliquots that were pipetted into plastic tubes. These tubes were marked, transported in special thermocontainers, frozen, and then stored at −80°C until analysis. The analyzed samples were subjected to one freeze/thaw cycle.

For plasma deproteinization, aliquots (10 µL) were mixed with 10 µL water (LiChrosolv; Merck KGaA, Darmstadt, Germany) and 80 µL methanol (Fluka, Munich, Germany) and incubated at room temperature. After 15 min, samples were centrifuged at 13,000 × g (MiniSpin plus centrifuge; Eppendorf AG, Hamburg, Germany) for 10 min. Deproteinized supernatants were then transferred to clean plastic Eppendorf tubes, and fifty volumes of methanol containing 0.1% formic acid (Fluka) were added to each tube. The resulting solutions were subjected to mass spectrometry analysis.

Samples were analyzed with a maXis hybrid quadrupole time-of-flight mass spectrometer (Bruker Daltonics, Billerica, MA, United States) equipped with an electrospray ionization (ESI) source. The mass spectrometer was set up to prioritize the detection of ions with a mass-to-charge ratio (*m/z*) ranging from 50 to 1,000 and a mass accuracy of 1–2 parts per million (ppm). Spectra were recorded in the positive ion charge detection mode. Samples were injected into the ESI source using a glass syringe (Hamilton Bonaduz AG, Bonaduz, Switzerland) connected to a syringe injection pump (KD Scientific, Holliston, MA, United States). The flow rate of samples to the ionization source was 180 μL/h, and samples were injected in a randomized order (e.g., control samples were run between case samples). Mass spectra were obtained using DataAnalysis version 3.4 (Bruker Daltonics) to summarize 1-min signals. Ion metabolite masses were determined from the mass spectrum peaks obtained using the DataAnalysis program. All peaks above noise level (signal to noise ratio >1) were selected, and the metabolite ion masses were pooled and processed using Matlab program (version R2019a; MathWorks, Natick, MA, United States). For the recalibration of all the peak *m/z* values, the internal standard losartan (m/z 423.169) was used.

Standardization of mass peak intensities was performed as described previously (Lokhov et al., 2020) by dividing the intensity by the standardization value, which was calculated for each peak separately as follows: the 50 Da range (which started 25 Da before and ended 25 Da after the *m/z* of the mass peak) was selected; all peaks inside the range were sorted in descending order according to their intensities; the intensity of the 150th peak was selected as the standardization value. Standardized intensities improved further analysis due to the correction of ion suppression of peak intensities (Lokhov et al., 2020). Standardized mass lists were normalized by applying the *normalize* function (which brings the sum of the intensities of the peaks in the spectrum to 1) of the Matlab program. The alignment of the *m/z* values of the mass peaks between different mass spectra was performed as described previously (Lokhov et al., 2011). The alignment algorithm used was previously specially developed and tested for the high-resolution mass spectra of blood metabolites obtained by DIMS and implemented as an iterative process based on the detection of correlation of mass spectrometry peak patterns.

### 2.3 Design of metabogram template for personal metabograms

**FIGURE 2:**
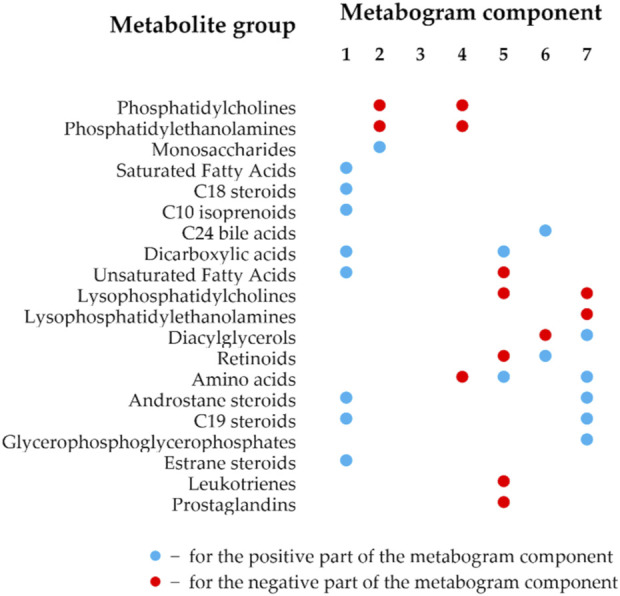
Composition of the clinical blood metabogram components. The composition of the metabogram components was measured by determining with which classes of metabolites they are enriched. Enrichment reliability expressed in *p*-values is presented in a previously published paper (Lokhov et al., 2023b).

The details of the metabogram construction using a reference cohort of healthy subjects are described in a previous study (Lokhov et al., 2023b). Briefly, DIMS was used to analyze blood plasma samples from 48 healthy people (reference cohort) to develop the metabogram template (Figure 1). The lists of mass peaks that were produced after mass spectra processing (alignment, standardization, and normalization) were analyzed using principal component analysis (PCA). The metabogram components were formed by the mass peaks corresponding to the highest positive or lowest negative coefficients (loadings) of the first seven principal components. The resulting sets of *m/z* values of mass peaks are presented in Supplementary Table S1. 70% of blood metabolome variance is explained by these sets of mass peaks, which were used in this study as a template to quickly produce personal metabograms. The composition of metabogram components (Figure 2) was determined by identifying the metabolite classes with which they are enriched. For this, MSEA was used (Xia and Wishart, 2010). Clinical blood tests (n = 71) were also used to determine the biological significance of the metabogram components (Lokhov et al., 2023b). Each metabogram component has two Z-score scales reflecting its measure, named the “positive” and “negative” parts, because the principal components involved in the development of the metabogram have both positive and negative coefficients (loadings). In short, the original variables that comprise the principal components are linear combinations of their coefficients. The structure of the data may be seen in the coefficients of each principal component. Larger positive or negative values indicate variables that contribute more to the component. *M/z* values corresponding to the highest and lowest coefficients of the first seven principal components—referred to the “positive” and “negative” parts of the metabogram components—were used to construct CBM reflecting the underlying data of the blood metabolome. In total, it amounted to about 5% each for the “positive” and “negative” parts of the detected peaks. The Z-score is a common way of representing data on a unitless scale and is the raw score minus the population mean, divided by the population standard deviation. With a normal distribution, the Z-score is connected to the *p*-values; for example, 1.64 corresponds to *p* = 0.05 (one-tailed), which is thought to be the cutoff for statistical significance and enables the detection of the sample’s deviation from the population. The metabogram’s Z-scores from −1.64 to +1.64 are considered to be in the normal range; up- and downregulation are represented by higher and lower Z-score values, respectively.

The components of the metabogram are formed by the functionally related metabolites of the blood involved in humoral regulation (component 1, called “regulatory”), lipid -carbohydrate metabolism (component 2), phospholypolysis (component 3, called “phospholipolytic”), lipid-amine metabolism (component 4), oxidized fatty acids (component 5, called “eicosanoid”), lipid intake into the organism (component 6, called “alimentary”), and liver function (component 7, called “hepatic”), thereby providing clinically relevant information. It should be noted that the identity of obtaining a CBM (sampling, sample processing, mass spectrometry, CBM design, and composition of metabogram components as presented in Supplementary Table S1), established in the first article that introduced the concept of CBM (Lokhov et al., 2023b) and further tested in subsequent studies, allows the obtained data to compare and relate the results obtained to the characteristics of the prototype of the same CBM-based LDT test.

### 2.4 Personal clinical blood metabograms

The study cohort (see Section 2.1), which included control individuals and patients with early-stage PD, was used to obtain personal CBMs. After standardization and normalization, the produced mass lists were aligned with the *m/z* values of the metabogram template (i.e., with 7 *m/z* sets corresponding to seven metabogram components; see Section 2.3). To obtain Z-scores of the metabogram components, the mass peak intensities belonging to the same metabogram component were converted into Z-scores and averaged (Lokhov et al., 2023b).

### 2.5 Cluster analysis

A cluster analysis was performed to give an overview of the metabograms of patients with the early clinical stage of PD. To do this, the *pdist* function (Matlab) was used to determine the Euclidian distances between the Z-scores of the metabograms’ components. The *linkag*e function created an agglomerative hierarchical cluster tree by calculating the distance between clusters using the “ward” algorithm. The *dendrogram* function was used to plot the dendrogram.

### 2.6 Diagnostic parameters

To assess the diagnostic potential of the metabogram for early clinical stage PD, the following diagnostic parameters were evaluated: sensitivity—the percentage of correctly identified positive results (the deviation is correctly assigned to metabogram component with Z-score out of normal range, i.e., Z-score < −1.64 or >1.64); specificity—the percentage of correctly identified negative results (the deviation from normal range is correctly not assigned to metabogram component with Z-score in normal range); and accuracy—the percentage of correctly identified positive and negative results.

The ROC curve was built by the *perfcurve* function (Matlab). The function also returned sensitivity and specificity values for diagnostics depending on the selected threshold Z-score value separating cases from controls and the optimal Z-score value for the highest diagnostic accuracy.

### 2.7 CBM signature of Parkinson’s disease

Considering the cluster analysis data and using the metabogram components that exhibit the greatest diagnostic power, a PD signature was formed. To confirm the inter-disease specificity of the PD signature, the ROC curves were built to separate control and patients with type 2 diabetes mellitus and obesity from control. Metabogram data for these subjects was taken from previous studies conducted on CBM research (Lokhov et al., 2023c; Lokhov et al., 2024). In the first case, the PD signature was directly applied to the metabogram data of diabetic patients. In the second case, the difference in the signature of obesity and PD, such as downregulation in the positive part of the first component of CBM at obesity, was additionally taken into consideration. Other differences between signatures were not considered since the absolute unspecificity of the PD signature for obesity was achieved.

## 3 Results

### 3.1 Studied subjects

Equally sized cohorts of patients and control subjects were obtained, aligned by gender and age, allowing for case-control comparison. Table 1 presents the clinical characteristics of the cohorts. The individual characteristics of the subjects are presented in Supplementary Table S2.

**TABLE 1:** Study cohort characteristics.

Characteristics	Values	
	Control subjects	Subjects with PD
Number	28	28
Age (years; mean ± s.d. (range))	62.8 ± 8.7 (45–77)	62.6 ± 8.6 (37–77)
Gender (males/females)	14/14	14/14
PD stages (1/1.5/2/2.5)^a^	—	6/6/12/4

^a^ PD, stages are according to modified Hoehn and Yahr scale (Goetz et al., 2004).

### 3.2 Metabogram data

**FIGURE 3:**
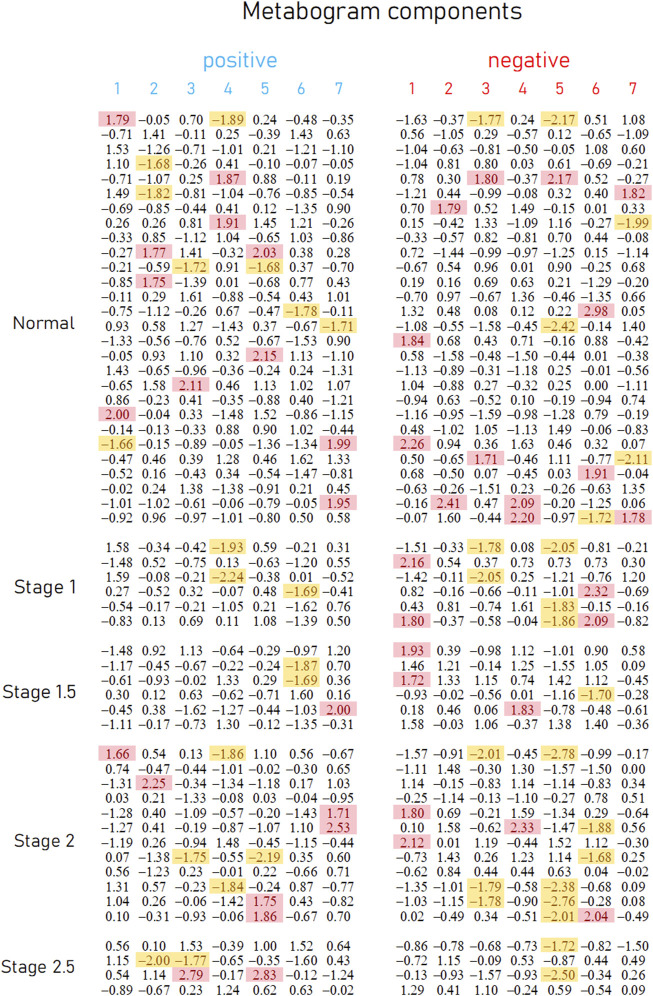
Metabogram data for control subjects and subjects with early-stage PD. Each row corresponds to the Z-scores of the metabogram components for an individual (components 1 to 7 for the “positive” and “negative” parts). Z-score is a measure of the metabogram components (from −1.64 to +1.64 is the normal range; up- and downregulation correspond to higher and lower Z-score values, respectively). Background color coding: red indicates upregulation in the corresponding metabogram component; yellow indicates downregulation in the corresponding metabogram component.

Mass spectrometry analysis, as the first analytical step of the CBM production (Figure 1), generated typical mass spectra of the low-molecular-weight fraction of blood plasma samples. On average, ∼9.7 thousand peaks were detected in the spectrum, which corresponds to the number of mass peaks in spectra used to design CBM (Lokhov et al., 2023b) and in other CBM-related studies (Lokhov et al., 2023a; 2023c; Lokhov et al., 2024). Aligned and standardized mass lists are presented in Supplementary Table S3. These mass spectrometry data were used to obtain personal metabograms for all subjects participating in the study (Figure 3).

**FIGURE 4:**
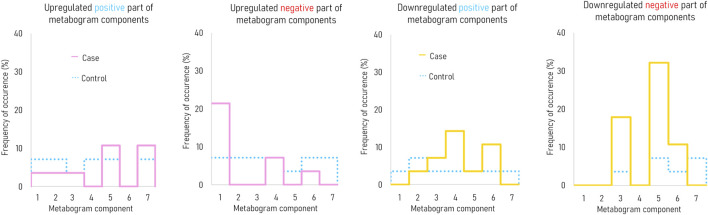
The frequency of deviations in the blood metabogram components for early-stage PD. The metabogram component deviates from the norm if the Z-score of the metabogram component is below −1.64 (indicating downregulation of the metabolites related to the metabogram component) or above 1.64 (indicating upregulation of the metabolites related to the metabogram component).

Figure 3 demonstrates that the components of the metabogram of PD patients deviate more frequently than those of controls, as evidenced by the frequencies of these deviations (Figure 4). Metabolites related to the negative components 3 and 5 and the positive component 4 are downregulated most frequently (Figure 4).

### 3.3 Statistical data and diagnostic parameters

The t-test results demonstrating the significance of the difference in the metabogram components in the case-control comparison are presented in Table 2. The difference for the negative component 5 is statistically significant (*p*-value 0.005), which indicates that PD-specific changes in metabogram can be attributed to the downregulation of the eicosanoids (Figure 2).

**TABLE 2:** Statistical significance of the deviation of the metabogram components in early PD (1–2.5 stages) from the control.

Metabogram Component		t-test (*p*-value^a^)
Positive	1	0.783
	2	0.939
	3	0.424
	4	0.082
	5	0.639
	6	0.129
	7	0.193
Negative	1	0.46
	2	0.499
	3	0.124
	4	0.184
	5	0.005^a^
	6	0.913
	7	0.760
All		0.180

^a^ *p*-value of statistically significant deviation from control.

To assess the diagnostic capabilities of the CBM, generally used diagnostic parameters were calculated. Table 3 displays sensitivity, specificity, and accuracy calculated based on the divergence of metabogram’s components from the normal range. The data in the table show the metabogram’s negative component 5 demonstrates the most diagnostic power for detecting the early clinical stage of PD with an accuracy of 62.5% (sensitivity of 32.1%, specificity of 92.9%) when a Z-score of −1.64 (corresponds to *p* = 0.05) is used to separate cases from controls. The lower diagnostic capability was demonstrated by the positive part of component 4 and the negative part of component 3, with diagnostic accuracy of 55.3% and 57.1%, respectively.

**TABLE 3:** Diagnostic parameters of the metabogram components for the detection of early-stage PD.

Metabogram component	Diagnostic parameters (%)		
	Sensitivity	Specificity	Accuracy
Upregulation of the metabolites (Z-score >1.64 for the metabogram components)			
Positive parts of metabogram components			
1	3.6	92.9	48.2
2	3.6	92.9	48.2
3	3.6	96.4	50.0
4	0	92.9	46.4
5	10.7	92.9	51.8
6	0	100.0	50.0
7	10.7	92.9	51.8
All (1–7)	4.6	94.4	49.5
Negative parts of metabogram components			
1	21.4	92.9	57.1
2	0	92.9	46.4
3	0	92.9	46.4
4	7.1	92.9	50.0
5	0	96.4	48.2
6	10.7	92.9	51.8
7	0	92.9	46.4
All (1–7)	5.7	93.4	49.5
Downregulation of the metabolites (Z-score < -1.64 for the metabogram components)			
Positive parts of metabogram components			
1	0	96.4	48.2
2	3.6	92.9	48.2
3	7.1	96.4	51.8
4	14.3	96.4	55.3
5	3.6	96.4	50.0
6	10.7	96.4	53.6
7	0	96.4	48.2
All (1–7)	5.6	95.9	50.8
Negative parts of metabogram components			
1	0	100.0	50.0
2	0	100.0	50.0
3	17.9	96.4	57.1
4	0	100.0	50.0
5^a^	32.1	92.9	62.5
6	10.7	96.4	53.6
7	0	92.9	46.4
All (1–7)	8.9	96.9	52.8

^a^ Metabograms component demonstrating the best diagnostic performance.

**FIGURE 5:**
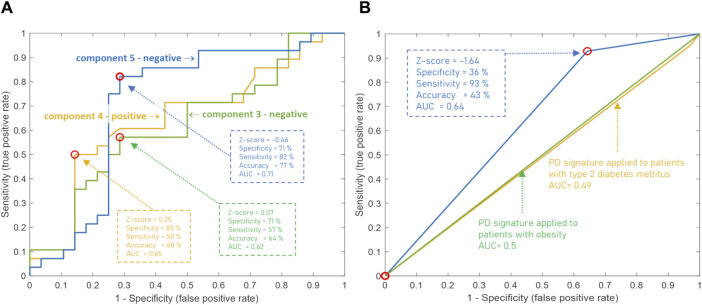
ROC curves based on the Z-score of the clinical blood metabogram (CBM) components for the diagnosis of Parkinson’s disease (PD). **(A)** ROC curves for the three metabogram components, which consist of the PD-specific signature: the negative parts of components 3 and 5, and the positive part of component 4. **(B)** ROC curve for the PD-specific signature, which consists of three metabogram components. Z-score −1.64 is used to distinguish between PD cases and controls. The PD signature was applied to patients with obesity and type 2 diabetes mellitus to show its inter-disease specificity.

The diagnostic potential of these components of the CBM was also assessed by building an ROC curve to determine the optimal threshold for separating cases from controls that provides the best diagnostic parameters. Figure 5A demonstrates that the accuracy of diagnostics was increased for the above-mentioned metabogram components to 76.8%, 67.9%, and 64.3%. This result confirms the diagnostic power of the CBM by a generally accepted method and shows that the Z-scores of the metabogram components can be further processed to improve diagnostic parameters.

**FIGURE 6:**
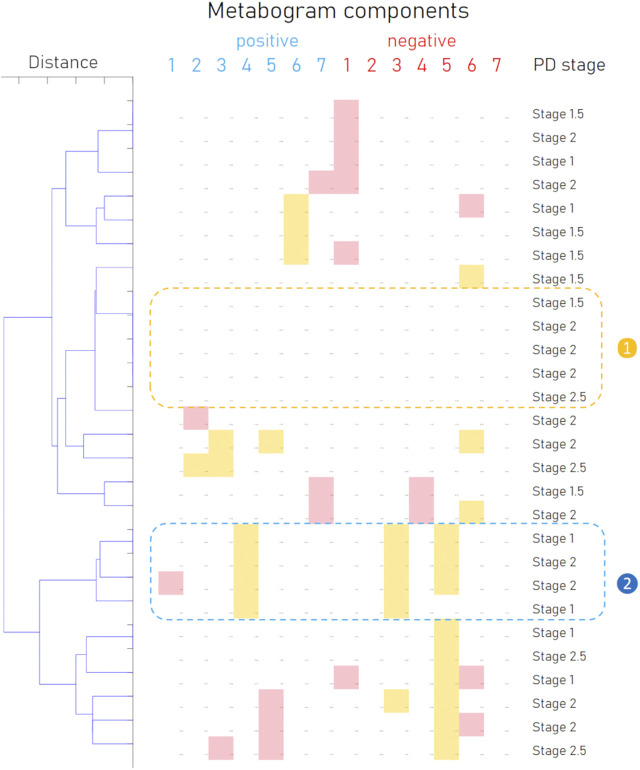
Cluster analysis of blood metabograms of PD patients involved in the study. Each row corresponds to the metabogram components for an individual (components 1 to 7 for the “ positive ” and “ negative ” parts). Color coding: red indicates upregulation in the corresponding metabogram component; yellow indicates downregulation in the corresponding metabogram component. The metabograms with no deviations ( ❶ ) in components and metabograms considered PD-specific ( ❷ ) are selected.

In addition to the fact that individual components of the metabogram are associated with PD, and some of them even have diagnostic power, the combinations formed by these components of the metabogram are also an important diagnostic feature—a signature of the disease. To identify such signatures, the patterns formed by deviating metabogram components were identified by cluster analysis (Figure 6). Clusters associated with stages of PD development were not revealed. One cluster, which can be seen as typical for PD, was created by various combinations of the most often deviating metabogram components (see cluster 2 on Figure 6). Therefore, it may be claimed that for a significant part of patients with early-stage PD, the CBM will show a PD-specific signature reflecting disease-associated metabolic alterations.

To confirm the inter-disease specificity of the PD signature, the ROC curves were built and compared with the ROC curves separating control from patients with type 2 diabetes mellitus and obesity (Figure 5B). The PD signature showed high sensitivity to PD at low specificity, while for other diseases the AUC was 0.49 and 0.5, which confirmed the inter-disease specificity of the PD signature. Therefore, if a PD signature is detected, early-stage PD is likely to be suspected. However, the absence of the PD signature does not exclude the underlying pathological condition. Perhaps the signature has not yet been formed at an early stage of disease in many patients, or it reflects only the dominant form of metabolic alterations in PD.

## 4 Discussion

The basic methods for diagnosing PD, a progressive degenerative condition of the central nervous system, are a medical history and a neurological examination (Jankovic, 2008). While effective treatment for PD depends on an early diagnosis (Gelb et al., 1999), a clinical diagnosis cannot be made until there is a large loss of dopaminergic neurons (Gibb and Lees, 1988). Moreover, the cost of the imaging of dopamine (Dopa) uptake efficiency diagnostic test based on positron emission tomography (PET) is high. As a result, a novel diagnostic laboratory test is needed. Biomarker discovery for such tests is hampered by PD’s ambiguous pathophysiology and complex character, and the use of panoramic techniques, as suggested, is more promising in this situation. Unfortunately, because of the consistency needed for clinical test registration, the clinical use of such ‘panoramic’ procedures, to which metabolomic analysis is related, is quite difficult. LDT usage gets around this problem. LDTs are defined by the Food and Drug Administration (United States) as tests that are created, produced, and used in the same laboratory (FDA, 2018). Therefore, the execution of metabolomics analysis in LDT format is sufficiently streamlined due to putting protocol development and standardization tasks under the purview of a single laboratory.

Although metabolomics, which measures the groups of metabolites that make up the metabolome, has been around for more than 20 years and the technologies it uses are nearly perfect, its application in medicine, even as LDT, is quite limited. Main cause of this is the preciseness of measurements, which allows for the precise measurement of numerous metabolites in a single run. Widely used in metabolomics, mass spectrometry techniques are typically capable of detecting hundreds of metabolites, which is essential for gathering biochemical data (Viant et al., 2017). Despite the use of cutting-edge mass spectrometry-based metabolomics technologies, the vast majority of the sample’s metabolites remain unknown (de Jong et al., 2017). Typically, only highly abundant and well-separated metabolites are identified. This is due to the difficulty of producing a clear mass spectrometric image of low-abundance metabolites, which constitute the majority of any metabolome. This means that the complexity of metabolomic measurements restricts the use of metabolomics in LDT format (Nalbantoglu, 2019; Lichtenberg et al., 2021; Lokhov et al., 2021).

The concept of the metabogram—a simplified single-subject metabolomics study—was developed to address this issue. The metabogram technique eliminates metabolite identification step (Lokhov et al., 2023b). Only groups of related metabolites are processed in the metabogram for this reason, and the use of MSEA (Xia and Wishart, 2010) quickly determines the enrichment of these groups with metabolite classes. As a result, group analysis takes the place of the challenging identification of individual metabolites. Additionally, data repeatability is improved by averaging metabolite data (peak intensities) within groups. For metabogram components, the coefficient of variation can be as low as 1.8% (Lokhov et al., 2023b), which is much lower than what is often found for individual metabolites (Crews et al., 2009). In order to validate the clinical utility of CBM for PD diagnosis, people with early PD were evaluated using CBM in this study.

According to the data obtained, it can be argued that, in terms of the frequency of occurrence and the joint appearance, PD-specific changes can be attributed to the downregulation of metabolites related to the eicosanoid component (negative part of component 5), the phospholipolytic component (negative part of component 3), and the positive part of component 4 (called the “phospholipid-amine” because of the co-directed changes in phospholipids and amino acids described by its negative part).

The most frequent deviation from the norm was revealed in the eicosanoid component of the metabogram (Figure 4). This deviation in patients with early-stage PD occurred 4.5 times more often than in the control group. A distinctive feature of this component of the metabogram is its enrichment with eicosanoids such as prostaglandins and leukotrienes. The close involvement of various eicosanoids in the development of PD can be read in the review by Chiurchiù V. et al. (2022). A variety of studies using both different biomaterials for research and the diversity of eicosanoids themselves led to the observation of multidirectional changes in their concentrations in PD. However, it can be argued that a group of eicosanoids in the blood decreases with PD (Zhang et al., 2021; Chistyakov et al., 2023) and even some eicosanoids exhibit neuroprotective effects (Rajan et al., 2020). Perhaps this group of eicosanoids is responsible for the decreased Z-score of this component of the metabogram.

The association of blood phospholipids with PD is an established fact and was already proposed for diagnostic purposes (Li et al., 2015). Oxidative stress is a significant factor in the onset and course of PD. Important elements of cellular membranes, phospholipids are essential for preserving the integrity and functionality of cells. Patients with PD have much higher lipid peroxidation products in their brains, which may be a connection between membrane damage and changes in phospholipid levels. However, it is possible that the detected changes in the phospholipolytic component may be due to a change in the concentration of phospholipids or may be associated with the activity of phospholipases. Previously, it was found that there is a link between phospholipases and PD (Mendez-Gomez et al., 2018; Wu et al., 2021). Thus, phosphatidic acid, a product of phospholipase PLD2 activity, is a second messenger in many cellular pathways and appears to be key to PLD2-induced neurodegeneration. The fact that α-synuclein is a regulator of PLD2 activity suggests that regulation of PLD2 activity may be important in the progression of PD.

Regarding downregulation reflected by the positive component 4, metabolites associated with it were not identified during the CBM design (Lokhov et al., 2023b). The list of molecular weights for which potential candidates exist was sparse and included several quasi-ions to which several elemental compositions corresponded (C_2_H_2_O_4_, С_4_Н_6_О_3_, С_4_Н_6_О_3_, С_5_Н_6_О_5_) (Lokhov et al., 2024). The elemental composition of C_2_H_2_O_4_ in the metabolite database corresponds only to oxalic acids, a degradation product of vitamin C, a deficiency of which in the body is associated with the development of PD (Brown, 2017). For the elemental formula C_4_H_6_O_3_, among the candidates are metabolites related to the butanoate metabolism pathway, such as acetoacetic acid (ketone body) and succinic acid semialdehyde. For C_5_H_6_O_5_, there is no alternative to oxoglutaric acid, which also belongs to butanoate metabolism. Interestingly, ketone bodies are associated with the development of PD, attributing neuroprotective properties to them (Maalouf et al., 2009). Moreover, the butadione metabolism pathway includes the formation of gamma-aminobutyric acid (GABA), and the connection between its level decrease and PD is well-known (Błaszczyk, 2016). Since the metabolites of this metabogram component were not reliably identified either according to metabolomics standards or during the design of a metabogram, the connection of this component with the butanoate metabolism pathway is hypothetical.

**FIGURE 7:**
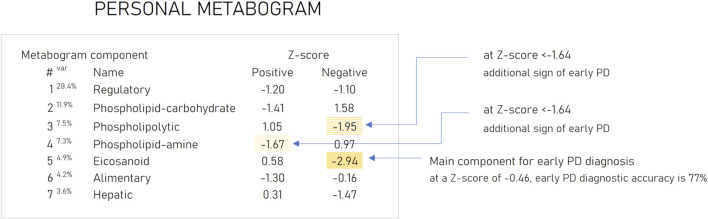
An example of the clinical blood metabogram. The percent of blood metabolome variance that the metabogram component explains is indicated by the superscript “Var.” The metabogram components are measured by the Z-score value, whose normal range is from −1.64 to 1.64. Higher and lower Z-scores are related to up- and downregulation of the blood metabolites corresponding to the metabogram component. The metabogram components most often deviated at PD are highlighted by background color. (Lokhov et al., 2023c).

Based on the results obtained, several types of metabograms can be distinguished in early PD (Figure 6). A metabogram without abnormalities, a metabogram with various non-systematic abnormalities that can be attributed to an individual’s disease course, or an individual health condition defined by other diseases, and a metabogram that can be attributed to a PD-specific metabotype. The last one manifests in the blood level of eicosanoids and is often associated with changes in the phospholipolytic and phospholipid-amine components. Figure 7 shows a metabogram in a simple format, showing the names of the components, the blood metabolome variance explained by each component, and the Z-scores of the components. The figure also provides a PD-specific signature – the components that contribute to the diagnosis of PD and can be potentially used to monitor the level of metabolic alterations during PD development and treatment. The inability to diagnose PD, as well as to monitor its course and the outcome of treatment in patients who do not have a PD-specific metabotype can be attributed to the limitations of CBM.

As said in the introduction, it's critical to remember that PD has a diverse pathogenesis involving a range of small molecular compounds. The specified metabolite composition of CBM components may cause some PD-specific metabolic alterations in the blood are undetected by CBM. Such alterations either do not have a significant effect on the main groups of blood metabolites reflected in CBM or refer to metabolites assigned to the remaining 30% of the variance of the blood metabolome not covered by CBM. However, modifying the CBM for a specific disease to increase its capabilities is beyond the scope of this work, which consists of testing a previously developed CBM design. The peculiarity of this approach is visible when comparing the results obtained with a previously conducted classical metabolomic study to search for biomarkers or a multimarker diagnostic signature of PD. In contrast to CBM, an AUC of 0.91 was achieved to diagnose PD in this single disease-focused study (Balashova et al., 2018). Contrariwise to such single-disease studies, the same CBM design applied to multiple diseases is more consistent with omics tests that identify changes at a significant portion of the metabolomic level, offering diagnostic capabilities for a variety of diseases.

From the described metabolic alterations, an additional feature of the PD signature can be suggested. The neuroprotective eicosanoids, whose reduction is reflected in the signature, not only inhibit neuroinflammation but also suppress oxidative stress (Tassoni et al., 2008). The change in the phospholipolytic component, as above indicated, can be caused by peroxidase oxidation and activation of phospholipase, which is recognized as an integral component of the oxidant stress response system (Adibhatla and Hatcher, 2008). The change in the metabolism of butadione led to the downregulation of ketone bodies with antioxidative properties (Kolb et al., 2021). Thus, the ketone body β-hydroxybutyrate is a direct antioxidant for hydroxyl radicals, an inhibitor of mitochondrial reactive oxygen species (ROS) production, and promotes the transcriptional activity of antioxidant defenses (Rojas-Morales et al., 2020). It can be assumed that the PD signature not only reflects the role of oxidative stress in PD development but also may indicate the risk of developing PD through the reflection of a reduced level of antioxidant activity in the organism. However, the confirmation of this assumption requires additional research.

The specificity of the identified signature to PD is also confirmed by its difference from previously published signatures of obesity and type 2 diabetes mellitus widespread in the population (Lokhov et al., 2023c; Lokhov et al., 2024). Moreover, when interpreting a PD-specific signature, the influence of the gut microbiota on CBM can be taken into consideration, the link to which was also described (Lokhov et al., 2023a).

The results of this study should also be assessed in the light of atypical parkinsonisms, the differential diagnosis of which from PD remains challenging. Although accurate diagnosis in the early stages of the disease plays an important role in prognosis and treatment strategy, distinguishing PD from, for example, parkinsonian-type multiple system atrophy (MSA-P) due to the similarity of symptoms can be difficult (Wenning et al., 1997; Goetz et al., 2004). The existence of the different clusters formed by metabograms in Figure 6 may be caused by MSA-P or other atypical parkinsonisms (progressive supranuclear palsy, corticobasal degeneration, and dementia with Lewy bodies). However, due to the rarity of their occurrence and the small cohort used, such a connection cannot be identified in this study, and this hypothetical statement rather serves as the basis for further research. Further studies in larger cohorts that include different parkinsonisms in sufficient numbers to obtain statistically significant data will demonstrate the potential of CBM in the differential diagnosis of PD and other parkinsonisms.

As for the clinical implications of the results of this work, an interpretation of the CBM can now be made for PD patients. This makes it possible to accurately analyze the metabolic changes in such patients and the dysfunctions of the body caused by these changes, relating or separating them from those specific to PD. Considering the pilot nature of the study, the feasibility of predicting the course of PD, assessing the effectiveness of treatment, and differentiating PD from atypical parkinsonism will become possible after additional research.

## 5 Conclusion

The measurement of the metabolome for clinical use is eagerly awaited and shows great promise. The metabolome, as its name suggests, is a level of organization of biological systems that is directly related to the global biochemical phenotype. One such attempt is the CBM, which, as demonstrated in previous studies, possesses the performance characteristics of a clinical test, and provides data that is clinically relevant. In this work, CBM was used to diagnose early PD, a condition that is very challenging to diagnose by laboratory testing, and its efficacy was verified. CBM allowed revealing a PD-specific metabotype, the measure of which not only provides diagnostic information but also opens up new opportunities to monitor PD progression and evaluate response to PD treatment.

## Data Availability

Publicly available datasets were analyzed in this study. This data can be found here: FigShare repository at https://doi.org/10.6084/m9.figshare.25487551.
